# Cost evaluation of temporary abdominal closure methods in abdominal sepsis patients successfully treated with an open abdomen. Should we take temporary abdominal closure methods at face value? Health economic evaluation

**DOI:** 10.1016/j.amsu.2020.06.007

**Published:** 2020-06-09

**Authors:** Alfredo Sanchez Betancourt, Gonzalez Cole Milagros, Pablo Sibaja, Luis Fernandez, Scott Norwood

**Affiliations:** a Centro de Investigación Clínica de Oriente, Universidad Estatal a Distancia, Sabanilla, Escazú, San Jose, Costa Rica; b Hospital Mexico, La Uruca, Costa Rica; c UT Health at Tyler, TX, USA

**Keywords:** Acute care surgery and trauma, General surgery, Open abdomen, Temporary abdominal closure, Economic health evaluation, Abdominal sepsis, Negative pressure therapy, Abdominal instillation

## Abstract

**Background:**

Many commercial and artisanal devices are utilized for temporary abdominal closure in patients being managed with an open abdomen for abdominal sepsis. The costs of materials required to treat patients with an open abdomen varies drastically. In Costa Rica, due to the lack of accurate information relating to the actual cost to manage a patient entails that the method with the least expensive materials is usually selected.

**Study design:**

A single-center retrospective review of 46 patients diagnosed with abdominal sepsis and successfully treated with an open abdomen and one of the three temporary abdominal closure methods during the year 2018 in a tertiary hospital was evaluated using a gross-cost pricing model developed by the authors. The three temporary abdominal closure methods were a locally manufactured Bogota Bag, and commercial abdominal negative pressure therapy dressing and negative pressure therapy with 0.9% saline solution instillation. The per-unit-costs were hospital day and intensive care day, number of surgical procedures per patient, cost negative pressure therapy kits.

**Results:**

Statistically significant cost reduction was observed in the cohort treated with negative pressure therapy with instillation as compared to the other temporary abdominal closure methods. The reduction of hospital length of stay, as well as fewer number of surgeries were the main contributing factors in diminishing costs. On average, the costs to treat a patient utilizing negative pressure therapy with instillation was nearly 50% lower than using the other two temporary abdominal closure methods.

**Conclusions:**

The costs relating to managing abdominal sepsis in the septic open abdomen vary greatly according to the temporary abdominal closure utilized. If the hospital length of stay, intensive care unit length of stay and number of surgeries required are the main parameters used in determining costs, the use of negative pressure therapy with 0.9% saline solution instillation reduces costs by nearly 50% in comparison to conventional negative pressure wound therapy and Bogota Bag. In this instance, the more expensive method at first glance, obtained a considerable cost reduction when compared to therapies that utilize less expensive materials.

## Highlights

• The costs of materials required to treat patients with an OA varies drastically. In Costa Rica, due to the lack of accurate information relating to the actual cost to manage a patient entails that the method with the least expensive materials is usually selected.
• Statistically significant cost reduction was observed in the cohort treated with negative pressure therapy with instillation as compared to the other TAC methods.
• On average, the costs to treat a patient utilizing negative pressure therapy with instillation was up to 50% lower than using the other two TAC methods.

## 1 Introduction

The use of the open abdomen in the management of severe abdominal sepsis has become a common therapeutic modality [1]. This treatment strategy has been utilized in various settings, such as abdominal compartment syndrome [2], severe pancreatitis [3], the need for revision (“second look”) surgery and abdominal sepsis [4] among others.

The decision of when to leave the abdominal cavity has been controversial in the past. Recently, guidelines have been proposed by the World Society of Emergency Surgery as to determine when and in what context should the abdominal cavity be left open. Regarding peritonitis, the following criteria were proposed: “*abbreviated laparotomy due to severe physiological derangement, the need for a deferred intestinal anastomosis, a planned second look for intestinal ischemia, persistent source of peritonitis (failure of source control), or extensive visceral oedema with the concern for development of abdominal compartment syndrome”* [5].

**Fig. 1:**
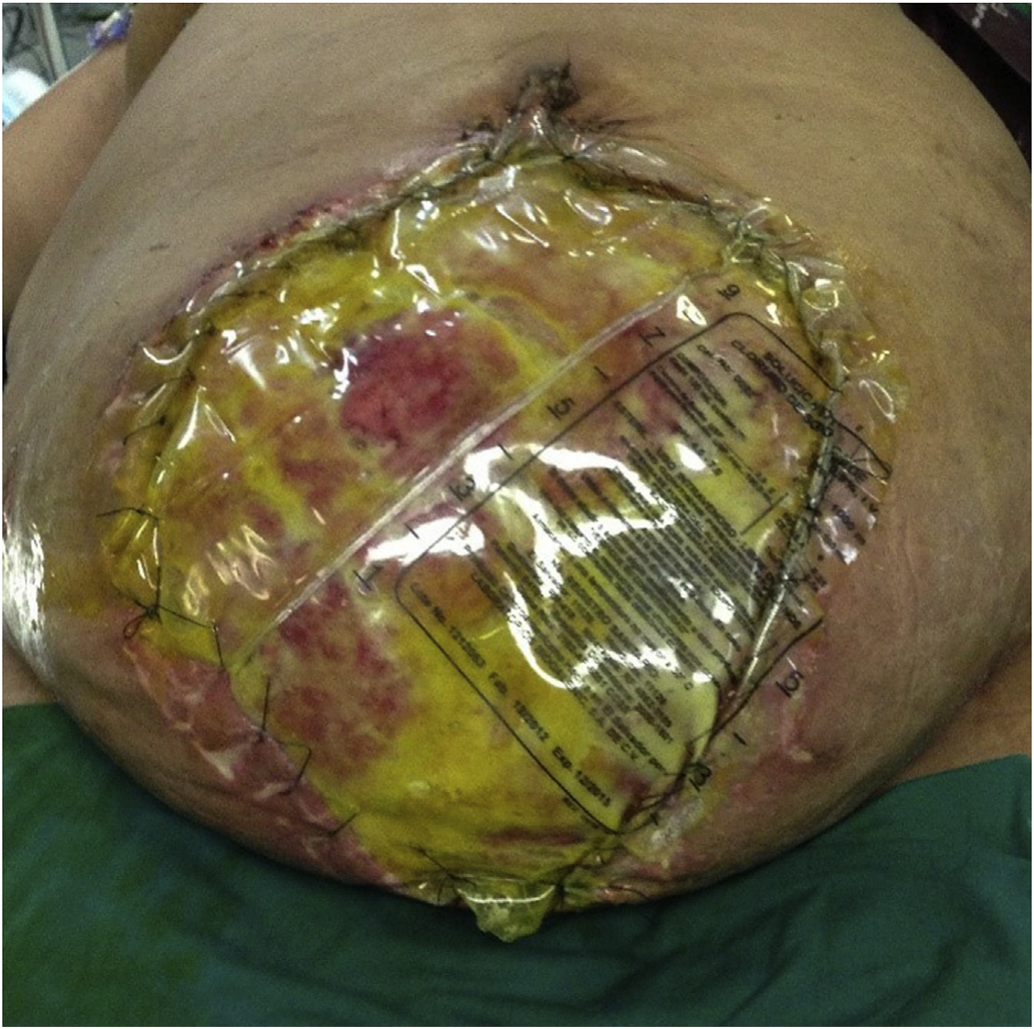
Locally manufactured Bogota bag placed in a septic abdomen.

Many types of temporary abdominal closure methods (TAC) have been described, including silo methods, such as the Bogota Bag(BB) [6] (Fig. 1), commercially fabricated Negative pressure wound therapy (NPWT) devices such as the ABThera™ dressing [7] and artisanal methods, such as Barker's Vacuum pack [8], as well as zipper and fascial closure devices [9].

**Fig. 2:**
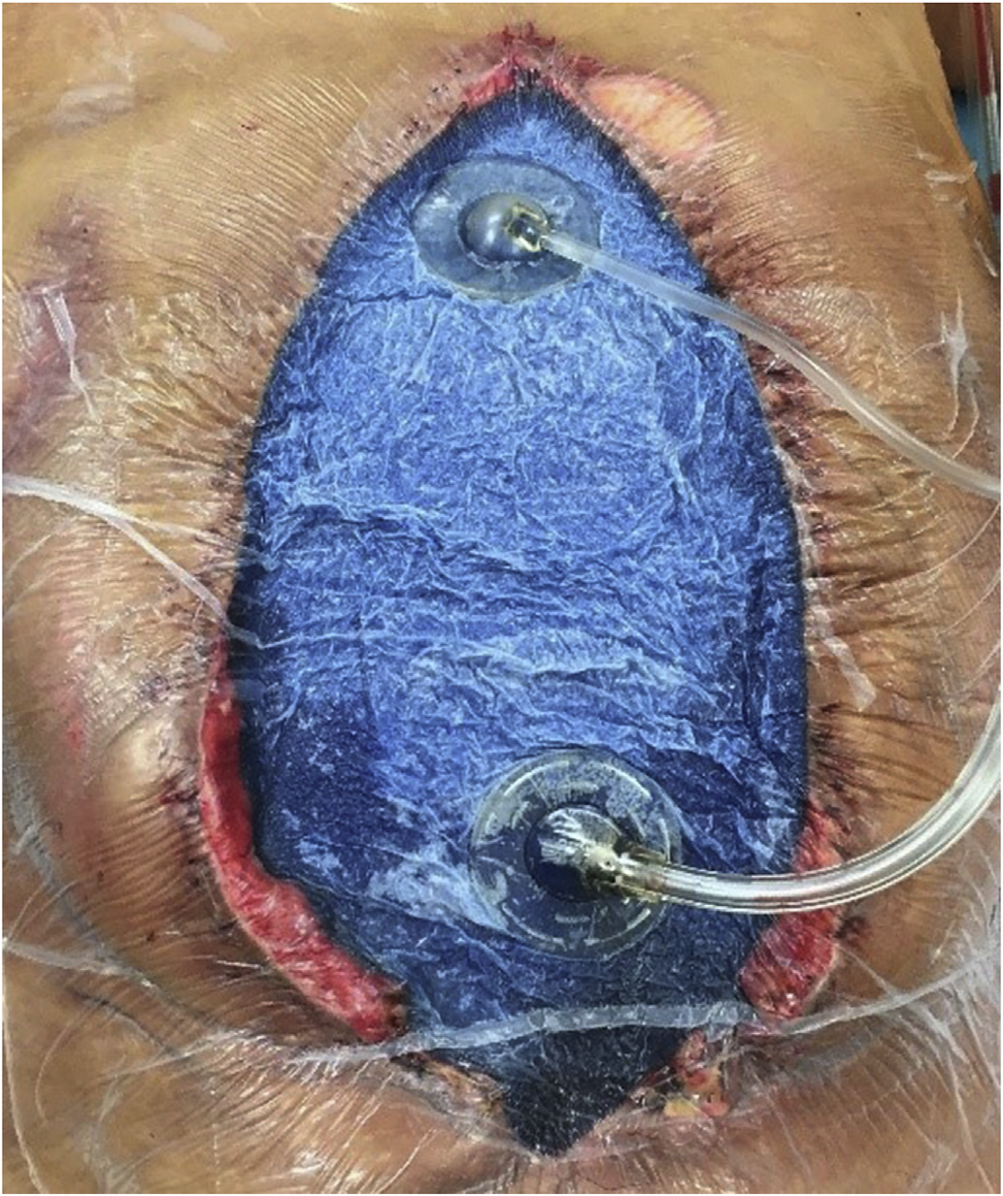
Negative pressure therapy with instillation provided via the ABThera™ negative pressure dressing with VeraFlow Therapy™.

The current data and expert opinions suggest that NPWT is the recommend method of TAC in trauma patients with abdominal compartment syndrome (ACS), the septic abdomen and other forms of abdominal surgical emergencies [10]. This method is currently the most popular type of TAC being applied worldwide [11]. A latter addition to the well-established NPWT devices has been the inclusion of fluid instillation into the abdominal cavity (NPT-I) in patients in which a continuous, noninvasive form of abdominal washout can be theoretically beneficial [12](Fig. 2).

In 2016, Sibaja, Sanchez et al. [13] published the first and largest case series to date in regards to the use of this method in patients with severe abdominal sepsis. Many other cases and case series have since been published regarding the instillation of various solutions into the septic open abdomen [[14], [15], [16], [17]]. Although there are various types of solutions instilled into the open abdomen, such as anesthetics [18], antibiotics [19,20] and antiseptics [20,21].The use of normal saline appears to be an acceptable alternative to other solutions in the septic open abdomen [22].

In Costa Rica, the public health system is funded through a tripartite payment modality, in which workers, employers and the government split the cost of health insurance, pensions and disability payments [22]. The public health system has had significant economic limitations [23], and cost reduction and system wide efficiency, has been prioritized within the various public hospitals. The “Caja” has been plagued with budget shortcomings and lack of funding [24], and has instituted a procurement method in which, usually, the lowest bidder frequently is selected. In this context, and due to the lack of quality data that would allow decision-makers to correctly gauge the costs of therapies being applied to various pathologies, the procurement of certain materials and therapies is usually driven by which item carries a lower up-front cost, limiting the use of certain therapies due to cost constraints. In the case of the surgical service where the study takes place, allocation of material resources is predominantly cost driven, therefore, the procurement of TAC methods that require a higher up-front cost, are sometimes unavailable to the surgical staff. In lieu of this, up-front costs tend to drive the decision making regarding what TAC is available to the clinicians. In this context is where this study takes place.

This study looks to ascertain the cost of treating patients with abdominal sepsis that require an open abdomen approach, and are treated with three different TAC methods, in hopes of determining if there is a cost difference with regards to the management of the same condition via three different treatment modalities, looking to determine the real cost to successfully obtain fascial closure and infectious control in patients with abdominal sepsis that require management with an open abdomen, with the intent of providing policy makers with an additional perspective as to which TAC method is most cost-effective.

## 2 Methods

A retrospective review of all patients that were managed with an open abdomen in a tertiary referral hospital in the capital city of San Jose during the 2018 calendar year was performed. Abdominal sepsis managed with an open abdomen is a relatively uncommon condition, therefore, we selected all patients that were diagnosed with this condition and managed with an open abdomen that met the initial selection criteria, as to have both a higher group of patients to analyze, as well as to be able to execute the study during a single fiscal year, which allows for a uniform cost analysis without having to adjust for inflation or other distorting factors. A Χ^2^ test of independence was performed on the data obtained to determine its validity. Data significance was set at p < 0.01.

Specific selection criteria were set forth for determining which patients were to be included in the study. The criteria for inclusion were:

• Patients undergoing treatment for abdominal sepsis that required management with an open abdomen with a Björck class 2 or higher [25].
• Patients treated with one of the three TAC methods used in the surgical services of the hospital (BB, NPWT & NPT-I).
• Patients surviving at least 72 h after being initially treated with an open abdomen and; not receiving more than one type of TAC method.
• Patients having successful fascial closure during the course of hospitalization.

A search of the hospital's electronic health records found a total of 84 patients that were managed with an OA technique, of which 46 met the inclusion criteria for this study. An Institutional Review Board (IRB) waiver was requested and obtained for this study.

A consolidated health economics evaluation statement (CHEERS) checklist was utilized as part of the health cost evaluation process [24].

**Fig. 3:**
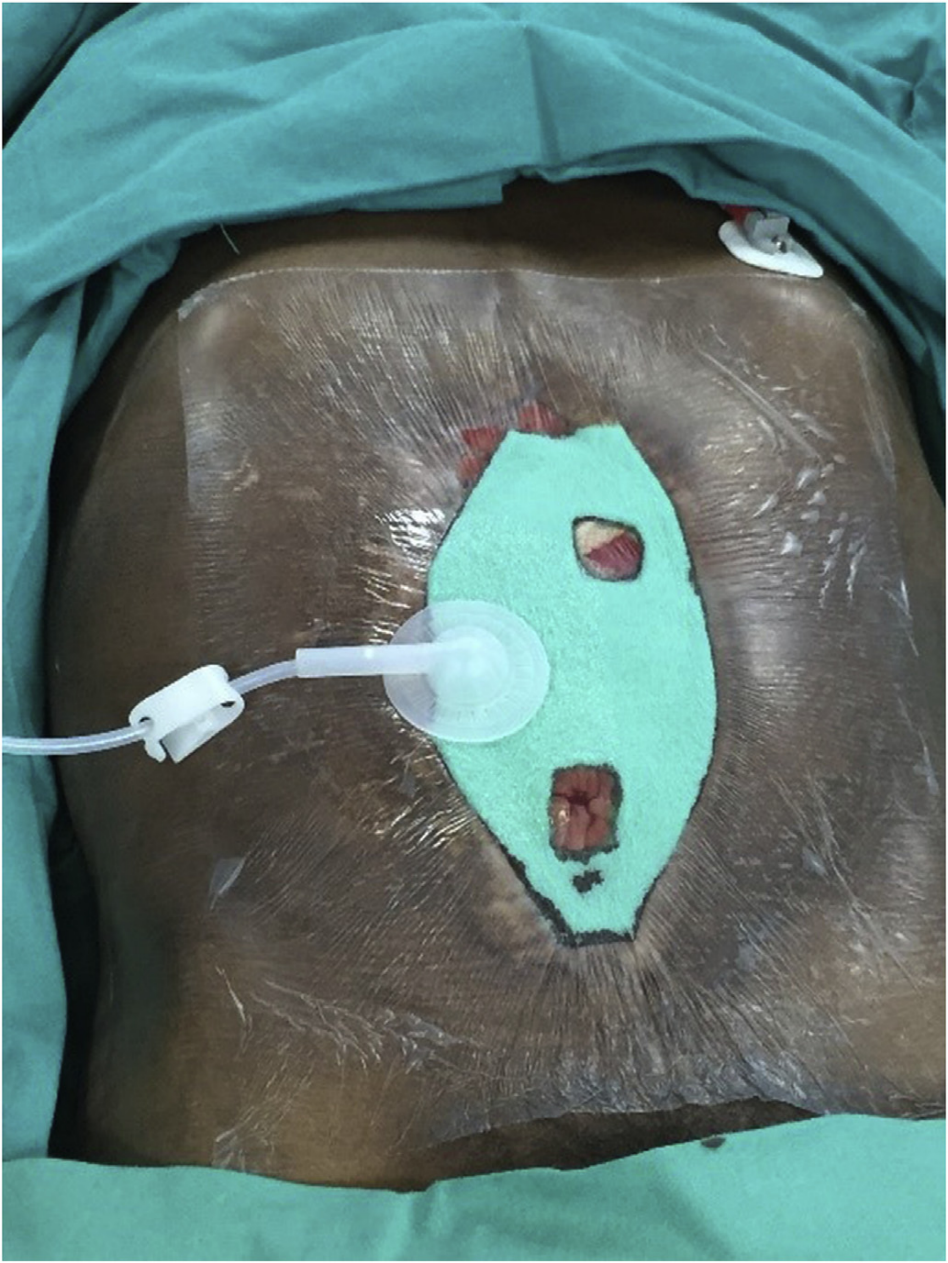
Genadyne™ negative pressure dressing applied to a septic open abdomen.

All patients treated with NPWT used the Genadyne™ abdominal dressing kit (Fig. 3.) The NPT-i group had their therapy delivered via KCI's ABThera™ dressing with VeraFlow therapy™ with 0.9% saline solution.

The patients were grouped according to the TAC method utilized. A gross cost model was used for this study, based mainly on the fixed costs the Costa Rica health service has. The cost of purchase of the individual items utilized to treat a patient with abdominal sepsis, as well as the man-hour costs, are priced into the bed-day costs. The parameters used were hospital length of stay (HLOS), which was composed of two parts: intensive care length of stay (ICULOS), and general surgery ward length of stay (GSXLOS). Additionally, the number of surgeries received (SX), including the final fascial closure procedure when performed, and the number of TAC kits (TACK) utilized for successful source control for were evaluated in the cost model. The HLOS is separated into two sections due to increased cost of stay per ICU day compared to a general surgery bed day. All lengths of stay are measured in days. No cost adjustment was performed.

The cost per analysis unit was determined utilizing the Caja Costarricense del Seguro Social's (CCSS) actuary evaluations that determine the *per diem* cost in both general and intensive care services, as well as the 1-h surgical rate that the institution has defined. The price for the commercial negative pressure dressings was obtained via the public contract bid in which the materials were acquired by the logistics department. All costs are shown in U.S. dollars (USD) using a conversion rate of 570 colones per USD.

The allocated costs in the July 2018 cost catalog of the CCSS were as follows: General surgery ward day: $1280.84. Intensive care day: $1478.42. One-hour operating room costs: $683.59. NPWT kit: $569.18. NPT-I kit: $711.98. This data is summarized in Table 1. The per patient cost was obtained via summation of the individual cost-unit consumption incurred in during the successful treatment of each patient. A discount rate was not applied to the values obtained because all economic data was gathered during the same 12-month period. No data adjustments were performed.

**Table 1:** Per item costs.

GSWD	$1280.84
ICUD	$1478.42
1h OR	$683.59
NPWT kit	$569.18
NPT-I kit	$711.98

GSWD: general surgery ward day. ICUD: intensive care unit day. 1HOR: 1-h operating room. NPWT kit: negative pressure wound therapy kit. NPT-i kit: negative pressure therapy with instillation kit.

The adequacy of utilizing an open abdomen in this patient group was not ascertained. The rationale as to why a TAC modality was selected in each patient is not discussed, although during the time of the study, not all TAC methods were readily available to the treating surgeons. Certain patient characteristics such as BMI, prior medical conditions, previous abdominal surgeries, nutritional status and other demographic information were not evaluated. All patients in this cohort were managed by the same group of surgeons.

## 3 Results

The total number of patients was 46, (19 females and 27 males). The average age of the cohort was 44.71 years of age ±SD 18.32 years. The most common diagnosis was perforated viscus (n = 11) followed by perforated acute appendicitis (n = 10). The third most common was anastomotic leak (n = 6). The most popular method of TAC was the Bogota bag (n = 20), followed by NPWT (n = 17) and the least common TAC method applied was NPT-i (n = 9).

Average hospital LOS for patients treated with a Bogota Bag (BB) was 29.3 days, NPT was 29.4 and NPT-I was 13.2. ICU LOS per TAC method was 9.5 days for BB, 22.11 for NPWT and 6.22 for NPT-i. Patients treated with BB received on average 3.75 surgeries. NPWT patients had 3.94 surgeries and NPT-I had 2.55 surgeries on average (*p* = 0.001).

The patients that received NPWT used 2.41 dressing kits and in the NPT-i group 1.33 kits were used (*p* = 0.001).

The most common diagnosis for BB patients was bowel perforation. In the case of the NPWT group and NPT-i group acute appendicitis with perforation (AAWP) was the most commonly detected condition respectively.

With regards to the total cost incurred to treat the patients included in the study; the total sum was $1,773,585.56‬ for the 46 patients. On average, BB patients cost on average $41,969.18(SE of mean = $6708.26) to treat. The NPWT group, which was the most expensive on average cost $43,614.75(SE of mean = $10,851.17) and the NPT-i patients had an average cost of $20,861.23 to treat (SE of mean = $3710.69).

The per TAC method groups are summarized in the following three tables (Table 2, Table 3, Table 4):

**Table 2:** Bogota Bag group characteristics.

BB group	
Age	44.45
Female	9
Male	11
HLOS	29.3
GSXLOS	19.8
ICULOS	9.5
SX	3.75
Kits	0
Cost per patient	$41,969.18
Total cost	$844,383.67
Most common diagnosis	Perforated bowel (n = 7)
	Trauma (n = 4)

**Table 3:** NPWT group characteristics.

NPWT group	
Age	45.64
Female	7
Male	10
HLOS	29.69
GSXLOS	7.58
ICULOS	22.11
SX	3.94
Kits	2.41
Cost per patient	$43,614.75
Total cost	$741,450.81
Most common DX	Acute appendicitis with perforation (N = 7)
	Pelvic abscess (N = 3)

**Table 4:** NPT-i group characteristics.

NPT-I group	
Average Age	43.55
Female	3
Male	6
HLOS	13.22
GSXLOS	7
ICULOS	6.22
SX	2.55
Kits	1.33
Cost per patient	$20,861.23
Total cost	$187,751.08
Most common DX	Acute appendicitis with perforation (N = 4)
	Anastomotic leak (N = 3)

Acute appendicitis with perforation sub-group:

A comparison of patients treated with an OA due to perforated acute appendicitis was performed, as to evaluate a shared etiology of abdominal sepsis in the treatment groups. AAWP was the most common cause of abdominal sepsis, comprising 24% of the diagnosis in the population evaluated. There were no OA patients managed with a BB for AAWP. The NPWT group had 7 patients with AAWP and the NPT-i had 4 (in both treatment groups it was the most common cause of abdominal sepsis). The NPWT group had an average of 39 years of age, an average HLOS of 12.8 days SD ± 7.56, 2.71 SD ± 0.69, surgeries per patient and an average cost of $19891.92 SD ± 10858.57.

The NPT-I AAWP patients had an average of 23.75 years of age SD ± 10.28, an average HLOS of 7.75 days SD ± 2.16, an average of 2 surgeries per patient SD ± 0 and an average cost of $12499.64 SD ± 3540.38.

## 4 Discussion

Multiple TAC methods have been described in the literature, and until recently, there were no clear indications as to which method was recommended and when a patient with abdominal sepsis should be have their abdomen left open [25]. The selection of TAC methods has been controversial and have often been determined by the surgeon's preference and the availability of the materials required to safely leave the abdominal cavity open. While there are many studies that look to validate one method over the other, the current consensus suggests using NPWT as the first line TAC method. The use of the ABThera dressing in conjunction with NPWT has yielded favorable results [26]. Instillation of normal saline [22], or other solutions, such as hypochlorous acid into the abdominal cavity(14) appears to provide an added benefit to patients who have abdominal sepsis that require management with an open abdomen [27]. The treatment of the OA with NPWT-i, offers an additional option with promising preliminary results in the treatment of patients with a septic abdomen [28]. While these early results are promising, additional studies should be conducted to confirm these initial findings.

Determining the true total cost of these surgical patients can be difficult. Various economic models appear to be unreliable [29]. The actuary model that determines the institutional fixed costs is subject to intense audit and is updated on a yearly basis. The reason why these cost units were selected is that the actuary model used to price in-hospital care integrates a large number of fixed expenses into the *per diem* cost, therefore, if the majority of economic resources are allocated into the bed-day cost, the increment or reduction of this particular cost-unit would drive the larger bulk of the cost-to-treat. The proposed cost model generated for this study, in our opinion, provides valuable economic guidance to decision-makers and hospital administrators. This mode of analysis will require further validation, to determine if it may be applicable in other settings, especially for those health care systems that do not have a large amount of fixed costs). The economic aspect of health care delivery is an important component, but not the only barometer by which to determine the utility of a given therapy. A healthcare technology assessment, requiring multiple criteria decision analysis [30] would be ideal to determine which TAC device is best suited for management of abdominal sepsis, both from a financial and patient outcome standpoint.

In developing countries, cost is the main barrier that limits access to surgery [31], therefore, the economic component of surgical strategies are of importance when determining the feasibility of a surgical procedure or method. In this context, it is the duty of the administrator to look for the most efficient and cost-effective way to obtain the desired results. The use of commercial negative pressure devices has been subject to significant analysis [32], where the clinicians are required to show the added value of specific technologies as a way to guarantee procurement of the materials required to perform certain procedures. As shown by Frazee et al., in 2013, the use of a commercial NPWT device, whose up-front costs were almost 20 times higher than that of an artisanal NPWT device, would likely incur in a $176,000 reduction in the management of 37 patients, and thus proved to be ultimately cheaper than the apparently less expensive option.

When a direct comparison of the underlying etiologies of abdominal sepsis was possible (as was the case with AAWP), the two different TAC methods used to manage this condition in the context of the OA in the cohort, NPT-i showed a cost reduction of 37.17% when compared to NPWT. This evaluation is consistent with the overall all-cause comparison between NPWT and NPT-i.

While not being directly addressed by this study, considering the reduction of up to 16 days in HLOS in the NPT-i group, the *per diem* wages that a patient would obtain once fit for work can also be factored into the final savings obtained via the method that most reduced the average stay of patients.

The analysis of patients that had successful fascial closure allowed for a direct assessment of a common patient outcome. Since the etiology of the abdominal sepsis varied from patient to patient, this cost model looks to compare dissimilar patients with one common link, all patients had their condition managed by an open abdomen successfully and were discharged with their abdominal incisions undergoing primary fascial closure. Morbidity and mortality were not part of the underlying objectives of this study, this data was not included in the study.

This study looks to evaluate some of the economic implications of various TAC methods in the open abdomen. Our focus is not to emphasize the clinical aspects of management with an open abdomen, but to determine the cost of patients that had been successfully treated with this approach.

## 5 Conclusions

In the study group there was significant variance in HLOS, ICULOS, number of surgeries and cost in the three TAC methods evaluated. The most significant variance lay in the NPT-i group, in which there was an on average reduction of 44.5% and 45.1% when compared to NPWT and BB respectively. The per patient cost needed to successfully treat patients with NPT-i diagnosed with abdominal sepsis was more than 50% less than those treated with the other two TAC closure methods evaluated. A large standard of error of the mean was observed in the final cost analysis.

Patients treated with BB and NPWT had very similar results with regards to cost per patient (NPWT was 3.8% higher). The most significant difference was ICULOS, that was 2.3 times higher in NPWT than BB patients.

The number of surgeries in patients treated with NPT-i was also reduced when compared to those treated with NPWT and BB, having received 1.29 surgeries less than those treated with the other two methods.

Even thought there was no age adjustment for patients or TAC method, there are multiple similarities both in age and morbidity (1.9-year difference on average between the youngest and oldest group of TAC methods). In the case of the NPWT and NPT-i group, the most common etiology of abdominal sepsis is the same (perforated acute appendicitis), therefore, we consider that even though there is a variance in the precipitating cause of the septic foci in the three TAC groups, there remains a fundamental similarity that allows for a valid comparison to be drawn. When an evaluation of patients with abdominal sepsis with the same etiology was carried out within the cohort, the results were similar to the overall group, showing a 37% reduction in costs when comparing the two negative pressure treatment modalities in patients with AAWP.

Based on economic data alone, NPT-i appears to be the more cost-effective treatment option compared to the other two TAC methods used to manage severe abdominal sepsis in our study population.

The limited scope of the study, as well as it being a single center and retrospective study, requires that further studies be undertaken to validate the conclusion reached by this report.

## Provenance and peer review

Not commissioned, externally peer reviewed.

## Declaration of competing interestCOI

Pablo Sibaja and Luis Fernandez are paid consultants for KCI, a company which manufactures TAC dressings and devices.

Alfredo Sanchez, Milagros Gonzalez and Scott Norwood have no conflicts of interest.

## Funding

No funding provided

## Author contribution

Conceptualization, A.S.B and M.G.C.; Methodology, A.S.B and M.G.C.; Investigation, A.S.B and M.G.C.; Writing – Original Draft, A.S.B and M.G.C Writing – Review & Editing, A.S.B, M.G.C, P.S.A., L.G.F, S.N.; Supervision, A.S.B.
